# Regulation of immune checkpoint blockade efficacy in breast cancer by FIP200: A canonical-autophagy-independent function

**DOI:** 10.15698/cst2020.08.229

**Published:** 2020-07-02

**Authors:** Syn Kok Yeo, Jun-Lin Guan

**Affiliations:** 1 Department of Cancer Biology, University of Cincinnati College of Medicine, Cincinnati, OH, 45267.

**Keywords:** FIP200/RB1CC1, immuno-therapy, checkpoint blockade, autophagy, TBK1

## Abstract

Immune checkpoint blockade (ICB) has emerged as a promising therapeutic strategy because of its potential to induce durable therapeutic responses in cancer patients. However, in the case of breast cancer, its application and efficacy has been limited. As such, combinatorial therapeutic strategies that can unlock the potential of ICB in breast cancer are of urgent need. In view of that, autophagy-related proteins that play a role in the autophagic cell recycling process have been implicated in the regulation of inflammatory and anti-tumor immune responses. Accordingly, autophagy-related proteins represent a group of prospective therapeutic targets in conjunction with ICB. In our recent study (Okamoto T *et al.* (2020), Cancer Res), we developed immune-competent mouse models of breast cancer which were deficient for the autophagic function of FIP200 or had FIP200 completely ablated to test the efficacy of ICB. We showed that although FIP200's autophagy function was required for progression of PyMT-driven mammary tumors, FIP200's canonical-autophagy-independent function was responsible for increased T-cell infiltration, IFN-signaling and ICB efficacy. These findings provide genetic proof of principle for a combinatorial therapeutic strategy that involves ablation of FIP200 to improve ICB efficacy in non-responsive breast cancers.

## DISSECTING THE ROLE OF AUTOPHAGY AND AUTOPHAGY-INDEPENDENT FUNCTIONS OF FIP200 IN BREAST CANCER

Although initial studies of autophagy genes in cancer implicated a tumor suppressive role, it has become apparent from studies in mouse models that autophagy genes also play important roles in promoting tumor progression and resistance to therapy. In parallel, there has also been an increasing appreciation for non-autophagy roles of autophagy genes and this has led to various efforts to specifically dissect the importance of autophagy in cancer, beyond complete ablation of autophagy genes. To this end, we have recently generated a FIP200 (RB1CC1) mutant allele which is deficient in binding ATG13 (referred to hereafter as FIP200 KI), leading to loss of function of the ULK1 complex that is crucial for the initial steps of autophagy. Assessment of this autophagy-deficient allele in PyMT-driven mammary tumors revealed that the ULK1-complex-functions of FIP200 were indeed essential for tumor development, growth and metastasis. This provides compelling evidence of the pro-tumorigenic roles of autophagy in cancer.

## FIP200 REGULATES THE TBK1-IFN SIGNALING AXIS IN A CANONICAL AUTOPHAGY-INDEPENDENT MANNER

**Figure 1:**
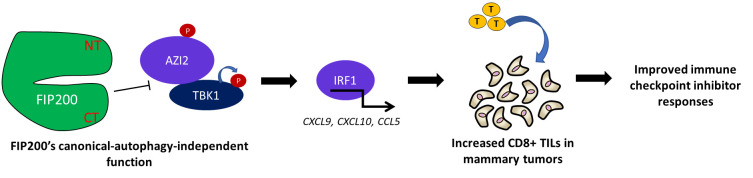
FIGURE 1: The canonical-autophagy-independent function of FIP200 regulates TBK1 activation, chemokine production, T-cell infiltration and immune checkpoint inhibitor efficacy. Schematic summarizing the phenotypes observed upon the loss of FIP200's canonical-autophagy-independent function and a working model of this regulatory process.

Interestingly however, we also found that the activation of TBK1 and downstream interferon (IFN) regulated genes were increased in mammary tumor cells, only upon complete ablation of FIP200 but not in cells carrying the autophagy-deficient FIP200 KI allele. Importantly, the changes in TBK1-IFN signaling was also associated with increased CD8^+^ T cell infiltration specifically in FIP200 ablated cells but not FIP200 KI tumors (**Figure 1**). The substantial deficiencies in tumor growth and progression that were induced by the FIP200 KI allele without significant activation of TBK1-IFN signaling led us to the interpretation that FIP200's non-canonical-autophagy function was responsible for limiting heightened anti-tumor immune responses. Mechanistically, we found that FIP200 could interact with the TBK1 adapters AZI2/NAP1 and SINTBAD but not TANK, which was in line with recent observations by Ravenhill and others from the Randow lab. We also observed that the phosphorylation of AZI2, in particular, was regulated in association with TBK1 activation by the non-canonical-autophagy function of FIP200.

While our results indicated that FIP200's autophagy-function within the ULK1-complex in tumor cells was dispensable in the regulation of TBK1 activation and CD8^+^ T-cell infiltration, it is worth noting that a number of studies have implicated the involvement of other autophagy genes such as ATG5 and ATG7 in the regulation of anti-tumor immunity. However, the distinct depletion of autophagy genes in the tumor versus the micro-environment may be factors that distinguish our observations from these other studies. It is also plausible that ULK1-complex independent autophagy programs were involved. Alternatively, it is possible that FIP200 and other ATG genes each have non-autophagy functions that regulate anti-tumor immunity. This notion is supported by recent observations of LC3-associated phagocytosis (LAP) in the regulation of immune-suppression. Since LAP involves autophagy genes such as ATG5, ATG7 and BECN1 but not FIP200, it represents a distinct non-autophagy function of these other autophagy genes that can impinge on anti-tumor immunity. Further work will be necessary to clarify the relative contributions of the autophagy gene network in the process of governing anti-tumor immunity.

## COMBINATORIAL TARGETING OF FIP200 WITH ICB INDUCES DURABLE RESPONSES IN ICB-REFRACTORY MAMMARY TUMORS

Robust durable responses and prolonged overall survival can be achieved via ICB but in the case of breast cancer, moderate responses are largely limited to triple-negative breast cancers (TNBCs) and HER2^+^ breast cancer. Since increased CD8^+^ T cell infiltration and activation of IFN-signaling have been reported to be associated with better ICB responses, we then evaluated ICB efficacy in PyMT mammary tumors that either carried the wildtype FIP200 allele, FIP200 KI allele or had FIP200 completely ablated. The MMTV-PyMT model has been reported to be of the luminal B breast cancer subtype. Consistent with the non-responsive nature of luminal B breast cancers to ICB, we found in our study that anti-PD1 in combination with anti-CTLA4 had no discernable therapeutic benefit in wildtype or FIP200 KI PyMT mammary tumors. Crucially, it was only in the tumors depleted of FIP200 that a substantial improvement in overall survival was achieved when anti-PD1 and anti-CTLA4 was administered in combination. Moreover, about 30% of mice in this cohort remained tumor free for up to 250 days after initial tumor appearance. Hence, we have unraveled a superior combinatorial therapeutic strategy that involves targeting the canonical-autophagy-independent function of FIP200 along with ICB, to achieve prolonged durable responses in mice bearing mammary tumors that were otherwise unresponsive to ICB. Altogether, our study provides genetic proof of principle, laying the groundwork for further understanding of the precise regulation of TBK-IFN signaling by FIP200. Ultimately, this could enable the development of therapeutics which target the canonical-autophagy-independent functions of FIP200, possibly in concert with inhibition of its autophagy function.

